# Chimpanzees can visually perceive differences in the freshness of foods

**DOI:** 10.1038/srep34685

**Published:** 2016-10-06

**Authors:** Tomoko Imura, Tomohiro Masuda, Yuji Wada, Masaki Tomonaga, Katsunori Okajima

**Affiliations:** 1 Department of Information Systems, Faculty of Information Culture, Niigata University of International and Information Studies, 3-1-1, Mizukino, Nishi-ku, Niigata, 950-2292, Japan; 2 Faculty of Human Sciences, Bunkyo University, 3337, Minami-ogishima, Koshigaya, Saitama, 343-8511, Japan; 3 Laboratory of Sensory Science, Food Research Institute, National Agriculture and Food Research Organization, 2-1-12, Kannondai, Tsukuba, Ibaraki, 305-8642, Japan; 4 Language and Intelligence Section, Primate Research Institute, Kyoto University, 41-2 Kanrin, Inuyama, Aichi 484-8506, Japan; 5 Faculty of Environment and Information Sciences, Yokohama National University, 79-7, Tokiwadai, Hodogaya-ku, Yokohama, Kanagawa, 240-8501, Japan

## Abstract

Colour vision in primates is believed to be an adaptation for finding ripe fruit and young leaves. The contribution of the luminance distribution, which influences how humans evaluate the freshness of food, has not been explored with respect to the detection of subtle distinctions in food quality in non-human primates. We examined how chimpanzees, which are closely related to humans, perceive the freshness of foods. The findings suggest that chimpanzees were able to choose fresher cabbage based on both colour and grey-scale images. Additional tests with images of novel cabbage, spinach, and strawberries revealed that one chimpanzee could detect the freshness of other fruits and vegetables. The critical factor in determining the judgements of freshness made by the chimpanzees was the spatial layout of luminance information. These findings provide the first known evidence that chimpanzees discriminate between images representing various degrees of freshness based solely on luminance information.

It is a critical but difficult task for most animals to engage in efficient foraging activities that ensure survival and reproduction. For instance, the availability of fruits and leaves, which are staple foods for many primates, often varies depending on the season^1^. Additionally, the nutritional quality of fruits and leaves varies according to their level of maturity^2^. To increase foraging efficiency, it is therefore necessary for animals to be able to appropriately identify the quality as well as the quantity of food.

In diurnal primates, which are closely related to humans, vision, in addition to chemical senses such as olfaction and gustation, provides essential information that drives food choice. In particular, the ability of humans and many other primates to determine colour is believed to be an adaptation for finding ripe fruit^3^,^4^,^5^,^6^,^7^ and young leaves^8^,^9^. Recent studies found that trichromatic colour vision may be advantageous when detecting sugar-rich fruits^10^.

On the other hand, recent studies in the domain of vision science reported that humans use luminance distribution in images to evaluate the freshness of wide varieties of food items such as fruits, vegetables, and fish^11^,^12^,^13^,^14^. For instance, Murakoshi *et al.*^13^ found that humans can adequately identify the level of degradation of individual food items by using the luminance distribution as a visual cue, even if the food items are observably different. These studies show that luminance can also provide important information, facilitating the perception of differences in the freshness of food. However, it remains unknown whether non-human primates can use the luminance distribution in an image to judge the quality of fruits and leaves.

In this study, we examined the extent to which chimpanzees are able to discriminate the freshness of the types of foods they eat daily. In Experiment 1 we investigated whether chimpanzees could discriminate between two images, each of which showed a cabbage leaf, but with varying degrees of freshness. Then we examined the invariants that contribute to the discrimination of freshness independently of colour (Experiment 2) and whether judgements of freshness are maintained when these factors are separated from the spatial structure (Experiment 3). In Experiment 4, we examined whether the discrimination abilities where generalisable by using other kinds of vegetable. We also analysed the effects of statistical information in the luminance distribution on the freshness judgements made by chimpanzees.

## Experiment 1: Freshness judgements involving novel pairs of images

### Method

#### Participants

Three chimpanzees (*Pan troglodytes*), a 35-year-old female named Ai, an 11-year-old male named Ayumu and a 31-year-old female named Chloe participated in all the experiments. These participants were living in the Primate Research Institute at Kyoto University with other group members in an enriched outdoor enclosure. They were not food-deprived and were fed fruits, vegetables, and monkey chow three times per day during the period of experimentation. The chimpanzees had previously engaged in various kinds of computer-controlled perceptual and cognitive tasks (e.g. refs 15 and 16). All experiments in the present study were approved by the Animal Care Committee of the Primate Research Institute of Kyoto University and by the Animal Research Committee of Kyoto University. The chimpanzees were tested and cared for according to the Japanese Act on the Welfare and Management of Animals and “The Guide for the Care and Use of Laboratory Primates, 3rd edition” issued by the Primate Research Institute of Kyoto University (2010).

#### Apparatus

We presented the visual stimuli on a 17-inch colour LCD monitor with a touch sensor (I-O DATA, LCD-AD- 171F-T) using a personal computer (Hewlett Packard Compaq, PM215AV). The resolution of the monitor was 1280 × 1024 pixels, and the viewing distance was approximately 40 cm. A piece of apple or a raisin were used for food rewards, which were delivered by a dispenser (Biomedica, BUF-310).

#### Stimuli

**Figure 1:**
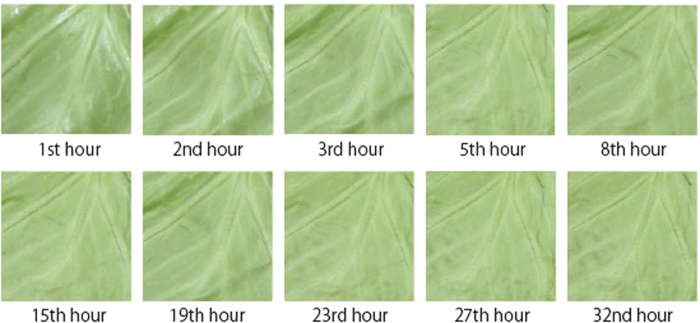
Examples of the stimuli used in Experiment 1. The number above each stimulus indicates the duration of degradation at the time the photograph was taken.

We used digital images of cabbage, which was also used in the experiment conducted by Wada *et al.*^14^. They took photographs of the degradation process of leaves of the cabbage using a digital camera with an automatic timer (Nikon COOLPIX P5100) which took a picture every hour for 32 hours (Fig. 1). The camera was affixed to a tripod stand in a box designed for taking photographs (D’ CUBE J; 116 × 100 × 100 cm). In the room where the pictures were taken, the humidity and temperature were kept constant at 6% and 30 °C respectively. The leaves were illuminated by two floor lamps with a colour temperature of 5400 K. The photographs were 3000 × 4000 pixels in size. As stimuli, we used 256 × 256 pixel sections of the pictures taken after 1, 2, 3, 5, 8, 15, 19, 23, 27, and 32 hours of the freshness degradation process. All images were cropped to the same approximate coordinates in Adobe Photoshop.

#### Procedure

The experiment consisted of training and test sessions. We used ten versions of the pictures described above. Five pairs (1 and 32, 2 and 27, 3 and 23, 5 and 19, 8 and 15 hours) were used in the training sessions. When the chimpanzees touched the start key at the centre of the touchscreen monitor, images from one of the pairs were displayed side by side. The chimpanzees’ task was to touch the image of the fresher cabbage out of the two alternatives. Once the chimpanzees had chosen one of the two images, the stimuli disappeared. A food reward and chime were given for the correct response. A buzzer sounded when the chimpanzee made an error. Following a trial where the chimpanzee made an error, a correction trial, where only the correct image was presented, took place. The correction trials were included in the methodology to keep the chimpanzees motivated.

First, the chimpanzees were trained using the pair of images with the largest difference in freshness, which were taken after 1 and 32 hours degradation. Once the average accuracy exceeded 90% in two successive sessions, a new pair of images with a smaller difference in freshness was adopted in the next session. Each session consisted of 30 trials. The training sessions continued until the criterion was met with all five image combinations, at which point the chimpanzees moved on to the test sessions. All three of the chimpanzees moved to the test sessions after a total of between 19 and 28 sessions (Ai: 28 sessions; Ayumu: 19 sessions; Chloe: 26 sessions).

During the test sessions, we examined the responses to 10 novel combinations using five original images (1, 2, 3, 5, and 8 hours) and an additional 10 novel combinations using the other five original images (15, 19, 23, 27, and 32 hours). The chimpanzees were required to choose the images of the fresher cabbage leaf. If the chimpanzees were able to discriminate between the two images based on the duration of the degradation process during the training sessions, they should be able to choose the fresher ones from new combinations of the images. A test session consisted of 42 trials (repeated six times each with five training pairs and two novel pairs). In the test trials using novel pairs, the participants were given a reward regardless of the choice of the image. Therefore, they could not learn during test sessions. The experiments took place over eight days, with the chimpanzees participating in five test sessions per day in order to cover all of the novel combinations within a day. For each novel pair, the chimpanzees completed 24 test trials in total.

For each chimpanzee, we calculated the proportion of correctly selected images. First, we conducted binomial tests to determine whether each chimpanzee chose the fresher images significantly more often than they would do at random (50.0%). Then we calculated the perceived freshness score (PFS) for each image using Thurstone’s method of paired comparisons to quantify the extent of the differences in the evaluations of freshness between two images^17^,^18^.

### Results and Discussion

**Figure 2:**
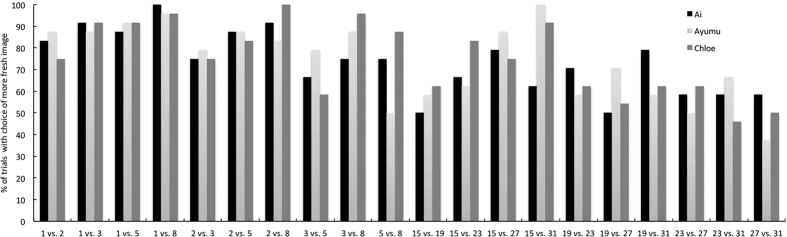
The percentage of trials in Experiment 1 in which the participants chose the image of the fresher cabbage leaf.

Figure 2 shows the proportion of trials in which each chimpanzee chose the image of the fresher cabbage during each of two test sessions. All three of the chimpanzees chose the fresher images from almost all of the pairs of photographs taken after 1, 2, 3, 5, and 8 hours. Across all combinations of 1 and 2 hours, binomial tests revealed that the chimpanzees chose the fresh images significantly more often than they would by chance, in which case they would select the fresher image 50.0% of the time (binominal test, *p* = 0.001). Most chimpanzees did not choose the fresher cabbage leaf for pairs of images taken after 3 vs. 5 hours of degradation significantly more often than chance, (Ai: 66.7% and Chloe: 58.3%) and 5 vs. 8 hours (Ayumu: 50.0%). Furthermore, all the chimpanzees failed to choose the fresher images in almost all pairs of photographs taken after 15, 19, 23, 27, and 32 hours. In the case of pairs taken after 15 vs. 27 hours, chimpanzees chose the fresher image significantly more often than they would have done had they been choosing at random (binominal test, *p* = 0.05).

**Figure 3:**
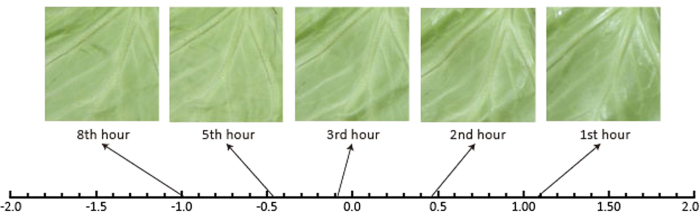
The yardstick of one-dimensional lines used for the perceived freshness score (PFS) for images taken after 1, 2, 3, 5, and 8 h of degradation.

The PFSs were calculated using Thurstone’s method of paired comparisons, which is typically used for sensory evaluation in humans. The proportion of fresher (or less fresh) images selected was calculated for all combinations of 1, 2, 3, 5, and 8 hours. Then the inverse function of the standard normal distribution was calculated and averaged for each image. Figure 3 shows the yardstick on which one-dimensional lines representing the PFS for each image are compared. A χ^2^ test revealed the level of agreement between the participants’ responses for each image (χ^2^ = 112.0, *p* < 0.01). As shown in Fig. 3, the PFSs decrease as the cabbage degrades. A one-way analysis of variance test (ANOVA) revealed a main effect of degradation time [*F*(4, 8) = 2.84, *p* < 0.01]. Ryan’s method indicated that there are significant differences between 1 h vs. 3; 5, 8 h, 2 h vs. 5, 8 h; and 3 h vs. 8 h (*p* < 0.05).

These findings suggest that the chimpanzees were generally able to choose the images of fresher cabbage from pairs of images that they had not been trained with; however, they failed to distinguish between images taken after 15 to 31 hours. This is consistent with human freshness rating scores for the same stimuli, which show that the differences in the freshness rating scores for images taken from 1 to 8 hours are larger than those for images taken from 15 to 32 hours^14^. These freshness ratings correspond to physical differences in colour that depend on the duration of degradation^14^. Hence, the discrimination performance of chimpanzees is generally consistent with freshness ratings provided by humans.

## Experiment 2: Freshness judgements based on achromatic images

We used achromatic versions of the original pictures to investigate the effects of chromatic information. Previous studies on humans have confirmed that the distribution of luminance contributes to judgments of freshness in cabbages and strawberries, even in grey-scale images^11^,^12^. If chimpanzees use the luminance distribution information to discriminate between images, as humans do, they should be able to distinguish differences in freshness from achromatic images.

### Method

#### Participants

The same three chimpanzees (Ai, Ayumu, and Chloe) that participated in Experiment 1 were tested.

#### Stimuli

The stimuli consisted of baseline and test stimuli. The baseline stimuli were five pairs of the original images (1 and 32, 2 and 27, 3 and 23, 5 and 19, 8 and 15 hours), which were identical to the training stimuli used in Experiment 1. Five pairs of grey-scale versions of the training stimuli were used as test stimuli.

#### Procedure

The procedure was identical to Experiment 1 except that the chimpanzees only participated in test sessions. The test sessions consisted of 36 trials (repeated six times each for five pairs of baseline stimuli and one novel pair of test stimuli). The chimpanzees’ decision was rewarded regardless of whether the image chosen was of the fresher cabbage. The chimpanzees participated over four days, with five test sessions per day, completing a total of 24 trials per pair of test stimuli.

### Results and Discussion

**Figure 4:**
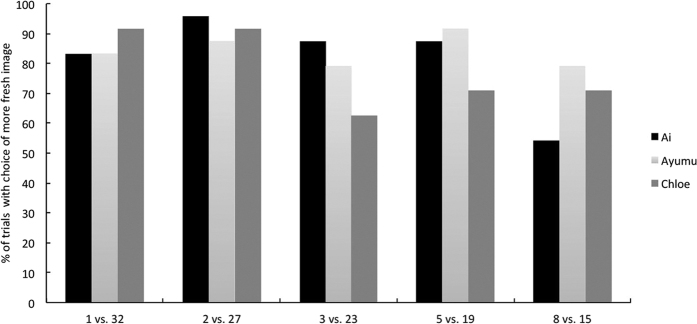
The percentage of trials from Experiment 2 in which the participants chose the image of the fresher cabbage leaf.

Figure 4 shows the proportion of trials for which each chimpanzee chose the image of the fresher cabbage. The chimpanzees chose the fresher images in more than 60% of trials in almost all combinations of grey-scale images. Binominal tests revealed that for all combinations, each chimpanzee chose the fresher image significantly more often than we would expect if they chose at random (binominal test, *p* = 0.001) except in the case of 3 vs. 23 hours, where Chloe was correct 62.5% of the time, and 8 vs. 15 hours, where Ai was correct 54.2% of the time.

These findings suggest that chimpanzees can distinguish the freshness of cabbages in achromatic images.

## Experiment 3: Effects of 3-D structure

Experiments 1 and 2 show that the chimpanzees could distinguish the freshness of cabbage in both colour and grey-scale images. However, it is possible that the chimpanzees discriminated between images in the pairs based on differences in luminance patterns, regardless of whether the image represented the surface of a cabbage leaf. Previous research on the human perception of glossiness suggests that the relationship between the 3-D structure of a surface and its pattern of highlights must be discernible for us to be able to judge what an object is made of ref. 19. If chimpanzees discriminate between images based on freshness, their performance should deteriorate when information regarding the 3-D structure of surfaces is absent. In Experiment 3 we created shuffled stimuli to examine the effects of 3-D spatial structural information on chimpanzees’ ability to determine freshness.

### Method

#### Participants

The three chimpanzees (Ai, Ayumu, and Chloe) that participated in Experiments 1 and 2 were tested.

#### Stimuli

The stimuli consisted of baseline and test stimuli. The baseline stimuli were identical to the training stimuli used in Experiment 1. The test stimuli were five pairs of new images created by randomising the positions of the pixels in the original images (e.g. ref. 20). The shuffled images contained the same colour and luminance information as the original images. However, spatial layout information was no longer present in these images.

#### Procedure

Only test sessions were performed. The test sessions consisted of 36 trials (repeated six times each for five pairs of baseline stimuli and one novel pair). The responses to the test stimuli were rewarded regardless of whether the image based on the fresher cabbage leaf was selected. The chimpanzees participated in four days of testing, with five test sessions per day. They completed a total of 24 test trials for each novel pair.

### Results and Discussion

**Figure 5:**
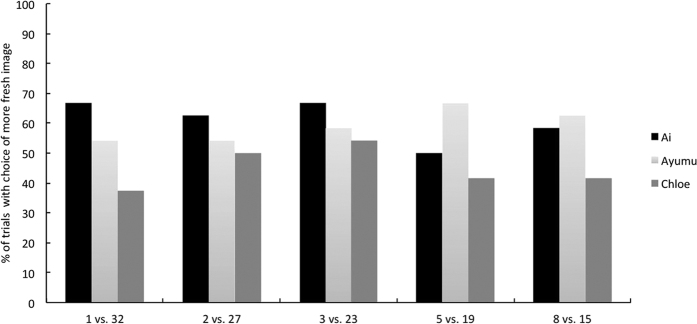
The percentage of trials from Experiment 3 in which the participants chose the image of the fresher cabbage leaf.

Figure 5 indicates the proportion of trials in which each chimpanzee selected the image of the fresher cabbage during testing. Binominal tests revealed that the chimpanzees did not select the shuffled images of fresher leaves significantly more often than they would at random.

The present findings show that chimpanzees discriminate between images based on luminance distribution only when images contain information pertaining to the 3-D structure of surfaces. This implies that luminance information of the spatial layout of surfaces also contributes to chimpanzees’ determination of the freshness of the cabbage in the images.

## Experiment 4: Do chimpanzees really represent “freshness”?

In the final experiment we examined whether chimpanzees can determine freshness in images of other kinds of fruit and vegetables. We used images of a different cabbage, spinach and strawberries to assess whether chimpanzees’ discrimination generalised to determining the freshness of other types of fruit and vegetables. We also analysed several statistical properties of the luminance distribution: the mean, standard deviation, skewness, and kurtosis. These properties were considered likely to be related to the judgments of freshness made by the chimpanzees.

### Method

#### Participants

The three chimpanzees (Ai, Ayumu, and Chloe), that participated in Experiments 1, 2 and 3 also participated in this experiment.

#### Stimuli

**Figure 6:**
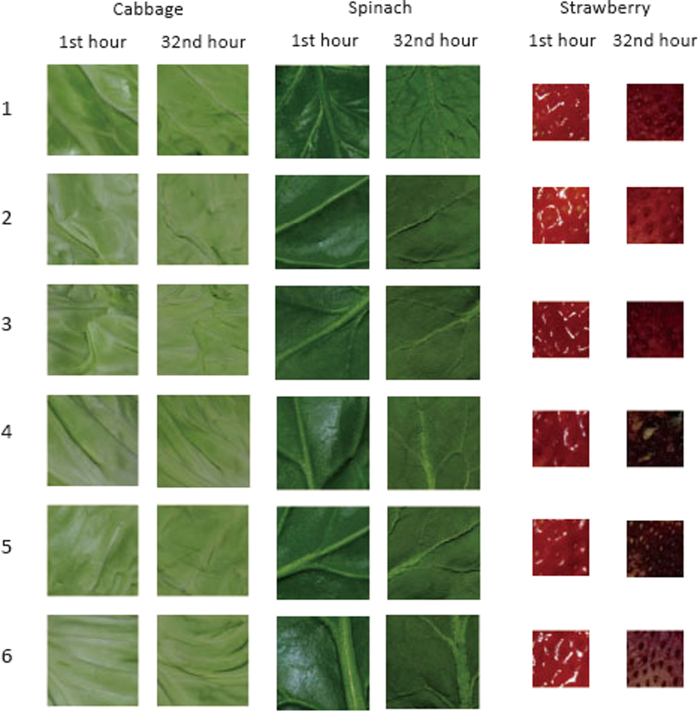
Examples of stimuli used in Experiment 4.

The stimuli consisted of baseline and test stimuli. The baseline stimuli were the five pairs of original images (1 and 32, 2 and 27, 3 and 23, 5 and 19, 8 and 15 hours), used as training stimuli in Experiment 1. The test stimuli were six different pairs of images of cabbage, which were different from those used in the training, and images of spinach, and strawberry taken after 1 and 32 hours of freshness degradation (Fig. 6). The method for taking the pictures was the same as the method used in Experiment 1. The sections of cabbage and spinach were 256 × 256 pixels in size, and the sections of strawberry were 128 × 128 pixels in size.

#### Procedure

As with Experiments 2 and 3, only test sessions were conducted. Three tests using the images of cabbage, spinach, and strawberry were conducted in separate sessions. Each test session consisted of 36 individual trials, which were repeated six times each for five pairs of baseline images and one novel pair. The experiments were conducted over a period of four days, with the chimpanzees undertaking six test sessions per day. They completed a total of 24 trials for each pair of test stimuli. Our hypothesis was that given that the chimpanzees learned to choose the images of fresher cabbage in Experiment 1, they may be able to distinguish freshness in new images of cabbage and other fruits and vegetables. To help understand the underlying mechanisms of chimpanzees’ freshness perception, we calculated the mean, standard deviation, skewness and kurtosis of the luminance distribution to examine the contribution they make to the discrimination.

### Results and Discussion

**Figure 7:**
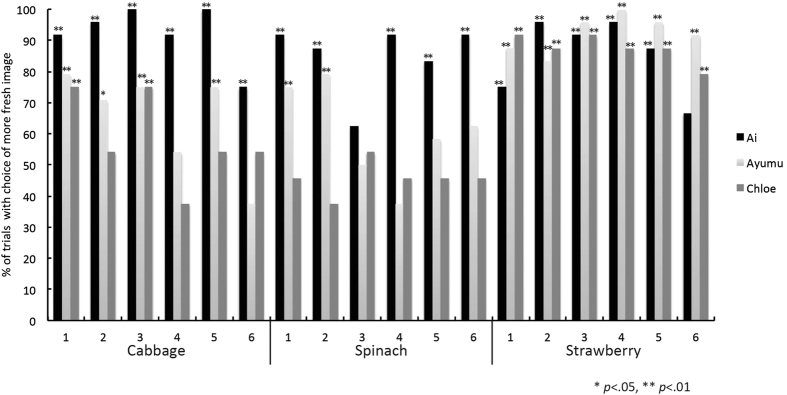
The percentage of trials from Experiment 4 in which the participants chose the image of the fresher cabbage, spinach, and strawberries during the test sessions.

Figure 7 shows the proportion of trials in which each chimpanzee chose the image of fresher cabbage, spinach, and strawberry. One out of three chimpanzees, Ai, consistently chose the images of fresher cabbage, spinach and strawberry. The performance of the other chimpanzees deteriorated when they were required to distinguish freshness in the images of cabbage and spinach. All chimpanzees were able to judge the freshness of strawberry with a high degree of accuracy. Although all of the chimpanzees were not necessarily able to distinguish the freshness of new vegetables and fruits in the image, these results show that part of the ability of freshness discrimination can be generalised and used to discriminate the freshness of other vegetables and fruits.

We conducted multiple linear regression analysis for each chimpanzee using differences in the luminance histogram parameters between the 1- and 32-h images used in Experiment 4 to determine how significantly various statistical properties of the luminance distribution contributed to the results. The adjusted R^2^ value in the model based on the mean, standard deviation, skewness and kurtosis was 0.42 for Ai, 0.83 for Chloe, and 0.74 for Ayumu. This indicates that for Ai, Chloe and Ayumu, respectively, 25%, 71% and 83% of the observed variation was explained by these four factors. The results with significant F ratios revealed that the regression models for all chimpanzees were unlikely to have occurred by chance (*p* < 0.01). Additionally, the partial regression coefficients of the mean and skewness of the luminance distribution in the images were significant for these two chimpanzees. For Chloe the mean coefficient value was −0.86, with *p* < 0.01, for Ayumu it was −0.77, with *p* < 0.01, and for Ai it was 0.59, with *p* < 0.05. In the case of skewness Chloe’s coefficient value was −0.39, *p* < 0.05, and Ayumu’s was −0.41, with *p* < 0.01. This implies that the mean and skewness of the luminance distribution contribute to the freshness judgment.

## General Discussion

The chimpanzees Ai, Chloe and Ayumu were able to distinguish differences in freshness in images of cabbage. Furthermore, they were able to choose images of fresher cabbage even when the images were converted to grey-scale (Experiment 2). However, their performance deteriorated significantly when the spatial layout was disrupted by randomly shuffling the positions of pixels, but the colour and luminance information were maintained (Experiment 3). These findings, alongside those of previous studies, suggest that the 3-D structure of surfaces of luminance information contributes to the judgment of freshness in both chimpanzees and humans. Although not properly demonstrated by all of the chimpanzees, the ability to discern freshness was generalised not only to images of a different cabbage but also to images of other kinds of fruit and vegetables (Experiment 4). These findings support the evidence that chimpanzees discriminate between images representing various degrees of freshness based solely on luminance information. The findings we present are consistent with previous studies that examined how humans evaluate freshness^14^ and extend recent comparative cognitive studies concerning non-human primates’ determination of food quality based on visual information^1^,^3^,^4^,^5^,^6^,^7^,^8^,^9^.

The results of Experiment 4 suggest that the mean and skewness of the luminance distribution contributed to the freshness judgments made by two of three chimpanzees. This result is consistent with studies of freshness perception in humans^11^,^12^,^13^,^14^. For instance, Wada *et al.*^14^ found that humans use the skewness of the luminance distribution in the judgment of freshness. They obtained this result by manipulating the values of the skewness in images where the luminance remained constant. Although it is difficult to make direct comparisons between our study and previous studies, as the experimental paradigm and analyses differ, it appears that human and non-human primates share the mechanism by which freshness is distinguished, based on statistical measures such as skewness. In the future, it will be necessary to clarify how the image statistics (e.g., mean and skewness) relate to freshness perception in both species.

Many studies have suggested that primates are typically able to discriminate colour, and especially that trichromatic vision is advantageous for finding ripe fruits and young leaves. Our study suggests that the statistical properties of the luminance distribution of images, such as mean and skewness, contribute to the ability to determine the freshness of food items. This is part of a small body of evidence that suggests that non-human primates use statistical information to distinguish differences in the surface quality of objects. Recent neurophysiological studies using a rich variety of images of materials to investigate how macaques determine the material of objects suggest that there is a strong correlation between neural responses and the statistical features of these images^21^. For instance, neurons that exhibit a selective response to glossy surfaces have been found in the macaque IT^22^. Texture-selective neurons related to material perception and categorisation have been found in the visual area V4 of the macaque brain^21^. These findings suggest that the ability to discriminate subtle differences pertaining to food quality and the material of an object is not unique to humans.

The results of the current series of experiments suggest that the chimpanzees were able to use information regarding luminance distribution and spatial layout to discriminate between images representing different degrees of freshness. However, this finding is subject to multiple interpretations. One possibility is that the chimpanzees discriminated between the images based on local visual features, such as patterns of highlights and shadows. Highlights and shadows are strongly related to the perception of freshness in humans^11^,^12^,^13^,^14^, and it may be difficult to determine whether the chimpanzees used these cues in the absence of perceiving freshness itself. The results of Experiment 1 indicate that the chimpanzees were able to discriminate between the images even when at least one highlight was present in each of the images (e.g., 1 h vs. 2 h). This provides evidence that highlights and shadows are powerful cues but that other cues may also contribute to the perception of freshness. A recent developmental study of material perception showed that human infants aged less than 5 months were able to detect slight differences in the illumination of images that were not detected by adults or infants over the age of 7 months^23^. Further research examining whether chimpanzees can discriminate between images involving different degrees of freshness taken under different light sources may help rule out this possible explanation.

Our findings do not necessarily demonstrate that chimpanzees understand the concept of “freshness” in the same manner as humans. To address this issue, future studies might investigate whether chimpanzees can pass a categorical matching task using images of various kinds of vegetables and fruits. If chimpanzees can evaluate the freshness of these images, they should be able to match the images based on their degree of freshness, even though they involve a variety of different vegetables and fruits. Other experimental approaches, such as exploiting the match between visual cues and cues from other sensory modalities involving real food based on their level of freshness, may provide evidence that chimpanzees perceive freshness. Further investigation of freshness perception in non-human primates may contribute to our understanding of the adaptive significance and evolutionary origins of human material perception.

## Additional Information

**How to cite this article**: Imura, T. *et al.* Chimpanzees can visually perceive differences in the freshness of foods. *Sci. Rep.* **6**, 34685; doi: 10.1038/srep34685 (2016).
